# Assessing the costs and environmental benefits of IMO regulations of ship-originated SOx and NOx emissions in the Baltic Sea

**DOI:** 10.1007/s13280-021-01500-6

**Published:** 2021-03-07

**Authors:** Sari Repka, Anne Erkkilä-Välimäki, Jan Eiof Jonson, Maximilian Posch, Janne Törrönen, Jukka Pekka Jalkanen

**Affiliations:** 1 Brahe Centre, Centre for Maritime Studies, University of Turku, University of Turku, 20014, P.O. Box 181, 28101 Pori, Finland; 2 Norwegian Meteorological Institute, Henrik Mohns plass 1, 0313 Oslo, Norway; 3 International Institute for Applied Systems Analysis (IIASA), 2361 Laxenburg, Austria; 4 Finnish Meteorological Institute, P.O. Box 503, 00101 Helsinki, Finland

**Keywords:** Atmospheric deposition, Baltic Sea, Maritime traffic, Monetary valuation, NOx, SOx

## Abstract

To assess the value of the environmental benefits of the Sulphur Emission regulation (SECA) that came into force in 2015, changes in depositions of SOx and NOx from ship exhaust gas emissions were modelled and monetized for the Baltic Sea region for the years 2014 and 2016. During this period, the total deposition of SOx in the study area decreased by 7.3%. The decrease in ship-originated SOx deposition from 38 kt to 3.4 kt (by over 88%) was translated into a monetary value for the ecosystem impacts of nearly 130 million USD, according to the EcoValue08 model. This is less than the modelled health benefits, but it is not insignificant. For NOx, there was no decreasing trend. The exceedance of the critical loads of SOx and NOx was also estimated. The effect of Baltic shipping on the exceedance of critical loads of acidification after SECA is very small, but Baltic shipping still has a considerable effect on the exceedance of critical loads for eutrophication.

## Introduction

In the Baltic Sea region (BSR), environmental degradation, such as acidification and eutrophication, has caused scientific and public concerns. In Europe, the emissions of key atmospheric pollutants have decreased steadily over the past decades (see EMEP 2018) due to the tightening of regulations on land-based sources. However, the Baltic Sea is one of the most heavily trafficked sea areas, and lately regulatory decisions to reduce airborne emissions from ships have been made (HELCOM 2018).

In January 2015, in accordance with the revised MARPOL Annex VI, the sulphur content of bunker fuel was not allowed to exceed 0.1% in the SECA (IMO 2008). Fuels with a higher sulphur content may also be used in combination with emission reduction technology that reduces the sulphur emission to levels corresponding to the use of low sulphur fuels. The Baltic Sea and the North Sea are also designated as NECAs (NOx Emission Control Areas). In NECAs, the aim is to reduce NOx emissions from shipping by 80% by using a three-tier system from January 2021 onwards (IMO 2017). TIER 1 came into force in 2005, and TIER 2 in 2011 with approximately a 20% reduction in NOx emissions from shipping compared with TIER 1 (IMO 2008). These two emission standards apply globally. The more stringent TIER 3, which came into force in 2016, requires an approximately 80% reduction in NOx emissions from TIER 1. Only gradual reductions of NOx emissions are expected as the NECA regulations only apply to new ships or major modifications of existing ships.

The depositions of SOx and NOx from the Baltic Sea shipping prior to and after the SECA regulation have been modelled with an atmospheric dispersion model (EMEP model; see Jonson et al. 2019). There were significant reductions in the SOx deposition, but there was no decreasing trend for NOx (Jonson et al. 2019). Approximately 15% of the NOx deposition in certain countries still originate from shipping (Jonson et al. 2019).

The abatement costs for the shipping industry were heatedly debated before the 2015 SECA regulation. It was estimated prior to the regulation that the health benefits of SECA regulation would exceed the costs (reviewed in EMSA 2010). To support planning and decision making, the cost efficiency of environmental regulations should also be estimated after the regulation comes into force. (Kalli et al. 2013; Lähteenmäki-Uutela et al. 2017; Åström et al. (2018).

Impacts on human health are of great interest in cost-benefit analyses (Im et al. 2018). In the case of the SECA, they have been evaluated both prior to the regulation (EMSA 2010) and after the regulation came into force (Barregård et al. 2019). In comparison, the benefits related to environmental improvements, such as the reduction of eutrophication and acidification, are not discussed as much (Ahlroth 2014) and in the case of SECA not evaluated in previous studies.

In this paper, we focus on the monetary valuation of changes in acidification and eutrophication by applying values that are available from the literature (Turner et al. 2004; Ahlroth 2014; Pizzol et al. 2015), as the monetisation of the environmental benefits of SECA regulation has not been done. Monetary valuation methods are controversial but useful as they provide more quantitative information than non-monetary methods by enabling easily understandable and comparable estimates of the costs of policy actions (Ahlroth 2014; Pizzol et al. 2015, 2017).

Critical load exceedance is another way of analysing the environmental effects of pollutants spatially on different scales. A critical load (CL) is defined as “a quantitative estimate of an exposure to one or more pollutants below which significant harmful effects on specified sensitive elements of the environment do not occur according to present knowledge” (Nilsson and Grennfelt 1988). CLs are calculated for terrestrial ecosystems and aquatic ecosystems, and a ‘sensitive element’ can be any part of an ecosystem, e.g. fine roots in forest soils or fish in a lake. We will analyse the effects of shipping on the exceedance of critical loads in the BSR before and after the 2015 SECA regulation. Critical loads (CLs) were originally derived in the context of acidification and are the limits for sulphur and nitrogen deposition, called CLs of acidity (CLaci). Later, limits for the eutrophying effect of N deposition have also been derived, i.e. CLs for eutrophication (CLeutN, also called CL of nutrient N).

## Materials and methods

### Modelling of SOx and NOx deposition

Atmospheric depositions of SOx and NOx have been calculated with the EMEP model rv4.14 with resolution of 0.1° × 0.1° as explained in Jonson et al. (2019). A detailed model description is available in Simpson et al. (2012). Model updates are described in Simpson et al. (2018) and references therein. All model runs have been made for three meteorological years: 2014, 2015, and 2016. In order to smooth meteorological variability, all the results presented here are based on the averages for these three meteorological years. Land-based anthropogenic emissions are from Eclipse version 5a (ECLIPSE V5 2014).

The evaluation of the exhaust emissions of marine traffic was based on the messages provided by the Automatic Identification System (AIS), which enables the identification and location determination of ships. The emissions are computed based on the relationship of the instantaneous speed to the design speed and technical information of the engines of the ships with a Ship Traffic Emission Assessment Model (Jalkanen et al. 2009). For the Baltic Sea, ship emissions for 2014 (pre 0.1% SECA) and 2016 (0.1% SECA) are used in the EMEP model calculations. For the remaining sea areas, ship emissions for the year 2015 are used, see Johansson et al. (2017). As the EMEP calculations are made for several meteorological years, monthly averaged emissions are used in this study.

### Spatial and temporal distribution of SOx and NOx deposition

**Fig. 1:**
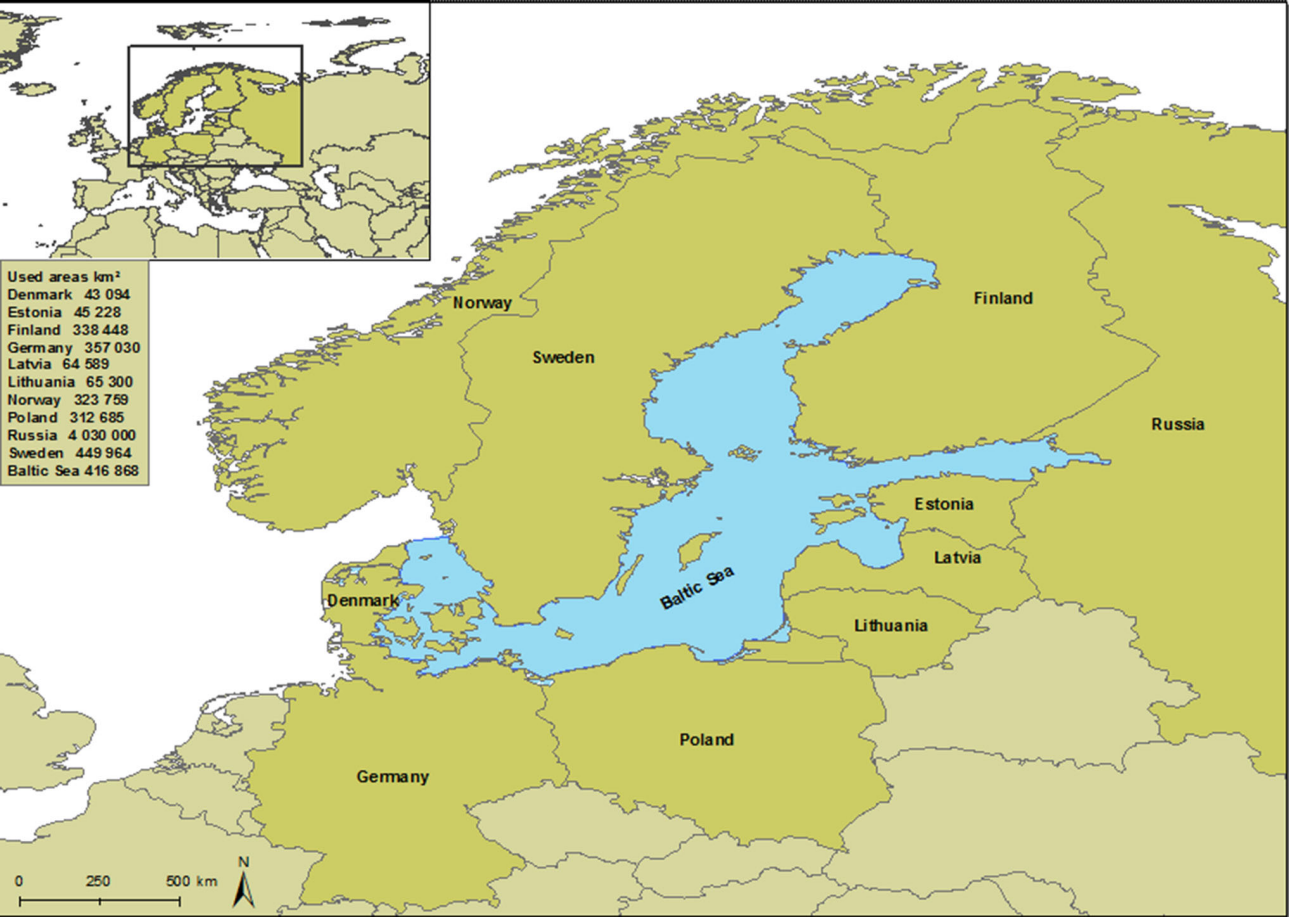
The areas of the riparian states and the Baltic Sea use in the calculations. The area of EMEP datasets covers larger area between 30° W, 45° E and between 30° and 75° N (approximately the area of the upper left corner map). The area of the Russian side (4 Mkm^2^) is presented in the smaller box of the upper left corner map. Geographic Coordinate System is World Geodetic System of 1984 (GCS WGS 1984)

The study area consists of the Baltic Sea and the riparian countries together with Norway (Fig. 1). The modelled deposition data of SOx and NOx include wet and dry deposition due to land and sea (ship) emission sources for the years 2014 and 2016. Depositions and spatial distribution maps were prepared using ESRI’s ArcMap 10.5.1. programme (ESRI 2017) in NetCDF format. The EMEP model datasets were in NetCDF format and covered an area between 30^o^ W, 45° E and between 30° and 75° N. The datasets were first converted into raster layers for ArcMap analyses. The ArcMap Zonal Statistics tool was used to create a table of the SOx and NOx deposition data values for the entire sea area of the Baltic Sea as one entity and for each riparian country, respectively (ESRI 2017). The total depositions of SOx and NOx for each country and for the sea area were calculated by multiplying the average deposition calculated with field calculator for the zone in question (mg/m^2^) by the zone’s total area (m^2^).

### Critical loads of acidification and eutrophication

Methods to compute CLs are summarised in Posch et al. (2015). Critical loads are calculated for terrestrial ecosystems (mostly forests) and for lakes and streams, but no methodology has been established to derive CLs for marine ecosystems. Critical loads are collected under the Convention on Long-range Transboundary Air Pollution (CLRTAP), hosted by the United Nations Economic Commission for Europe (UNECE, www.unece.org/env/lrtap), and used to support European assessments and negotiations on emission reductions (Reis et al. 2012; EEA 2014; De Vries et al. 2015; Maas and Grennfelt 2016).

If a deposition is higher than the CL at a site, the CL is said to be exceeded. For CLeutN, the exceedance is the difference between total deposition of N and CLeutN (set to zero, if negative). For acidity, the exceedance is a well-defined distance measure from the critical load function (see Posch et al. 2015). To obtain a single exceedance number for a grid cell (or any other region), the so-called average accumulated exceedance (AAE) is used, defined as the weighted mean of the exceedances of all ecosystems within the grid cell, with the weights being the respective ecosystem areas (Posch et al. 2015). The current European CL database for acidity and eutrophication CLs was used (Hettelingh et al. 2017).

Model calculations for all three meteorological years were made for the following five scenarios:

- ‘Base 2016’: All emissions from 2016/2015
- ‘No Balt’: No Baltic Sea ship emissions, elsewhere 2016/2015 emissions
- ‘Balt 2014’: 2014 Baltic Sea ship emissions, elsewhere 2016/2015 emissions
- ‘Baltic Sea 2030’: 2030 Business-As-Usual (BAU) emissions.
- ‘No Balt 2030’: No Baltic Sea ship emissions, elsewhere BAU 2030 emissions

### Monetary valuation

To evaluate the economic effects of SECA in the BSR, monetisation models for atmospheric pollution were reviewed from the literature. In many cases, the monetary values are based on the surveys of individuals’ willingness to pay for environmental quality, while some are based on the prevention or restoration costs (e.g. Ahlroth 2014, Pizzol et al. 2015). Economic modelling requires a number of simplifying assumptions and value choices depending on the scope, societal context, and geographical area that the models are made for (Turner et al. 2004; Ahlroth 2014; Pizzol et al. 2015). Results may not be statistically robust due to small sampling size or weights may be based on the old models (Turner et al. 2004). Therefore, the results of different weighting factors should be used bearing in mind that the results are more indicative than absolute values (Ahlroth 2014; Nguyen et al. 2016).

According to Turner et al. (2004), the receptors of environmental impacts of air pollution include the categories of buildings, agriculture and ecosystems, as well as climate change. There are relatively few studies on costs for environmental impacts compared to human health effects, and they mostly originate from 1990s and 2000s. However, the weighting sets do not always cover all the environmental impacts, e.g. the impacts of acidification (Ahlroth and Finnveden 2011). We applied values of sulphur and nitrogen from three weighting sets, i.e. STEPWISE2006, Ecovalue08 and Eco-cost/Value Ratio (EVR), and from the review of Turner et al. (2004) summing several values of external costs and benefits of waste management to one mean value (see Table 1; Turner et al. 2004; Weidema 2009; Ahlroth & Finnveden 2011; Vogtländer & Bijma 2000). The weighting factors have been calculated for atmospheric emissions, but we apply them to depositions, assuming that the direct environmental impacts to ecosystems, agriculture and buildings are mostly generated in the deposition processes.

**Table 1:** Monetary values of the environmental impacts of SOx and NOx. Letters refer to the calculations of the monetary values in Tables 6 and 7. The values provided by Turner et al. (2004) are compared with Stepwise2006 by Weidema (2009) using the exchange rate of 1.45 EUR/GBP in 2003. The same EUR/GBP exchange rate is used for all values of Turner et al. (2004). Inflation is calculated to April 2018 and converted to USD using the April 16^th^ 2018 currency rate of 1 EUR = 1.237 USD

Substance	Impact categories (source in brackets)	References	Monetary values of environmental impacts	Monetary values of environmental impacts USD_2018_ kt^−1^	Reference to Tables 6 and 7
SO_2_	Ecosystem impacts (1)	Weidema 2009	150 EUR_2003_ t^−1^	235 550	A
SO_2_	Impacts on agriculture (2) Impacts on buildings (2) Ecosystem impacts (2) TOTAL OF SO_2_ impacts	(2)Turner et al. 2004, Weidema 2009	20 838 12 870 EUR_2003_ t^−1^	1 366 198	B
SO_2_	Acidification (3)	(3) Ahlroth & Finnveden 2011	30 SEK_2010_ Kg^−1^	3 864 900	C
SO_*x*_ equivalent	Prevention of acidification (4)	(4) Vogtländer & Bijma 2000	6.40 EUR_1999_ Kg^−1^	10 968 011	D
NO_*x*_	Ecosystem impacts (1) Agricultural impacts via photochemical ozone (1) Impacts on buildings (2) Fertilisation effects (2) TOTAL OF NOx impacts		600 400 300 Benefit of 200 1 100 EUR_2003_ t^−1^	1 727 376	E

We assumed that both sulphate and SO_2_ used in the valuation by and large indicate the environmental impacts of sulphur. STEPWISE2006 also includes values for ecosystem effects of SO_2_, which we applied here (Weidema 2009; Table 1, Pizzol et al. 2015). Furthermore, we used the mean values of the impact on agriculture and buildings from the summary of values of key pollutants compiled by Turner et al. (2004) from 10 previous studies. Ecovalue08 was developed for the Swedish environment and adapted for Scandinavia (Ahlroth and Finnveden 2011), and thus, it is a geographically fitting set for the sea area and riparian countries of the BSR. The highest values for SO_2_ in terms of prevention of acidification are produced by the Eco-Cost/Value ratio (EVR) (Vogtländer and Bijma 2000; Vogtländer et al. 2001). EVR is based on abatement costs. We used these models to estimate the damage in monetary values of ship-originated sulphur deposition on the sea area of the Baltic Sea and its riparian countries.

Values for NOx monetisation are available from STEPWISE2006, and the summary values are compiled by Turner et al. (2004). NOx values for different impact categories were summed to one single value representing the monetary value of NOx deposition. The calculation of the monetary values of SOx and NOx deposition from Baltic Sea shipping was done by multiplying the deposition of SOx and NOx with the monetary values. The monetary values were inflation adjusted, according to the currency of April 2018 and then converted to US dollars (USD).

The results of depositions of SOx and NOx from Baltic shipping to the sea area and riparian countries are also discussed in Jonson et al. (2019). In our study, we have used the same data as basis for the monetisation and critical load exceedance calculations. The monetisation models include all the impact categories of acidification and eutrophication, thus, also the effects of the exceedance of critical loads.

## Results

### Depositions of SOx and NOx from shipping

SOx deposition originating from the Baltic Sea shipping was 2.2% of the total deposition in the study area in 2014. In 2016, the share of ship-originated SOx deposition had decreased to 0.3% (Table 2, Jonson et al. 2019). The share of the ship-originated SOx differed between countries due to factors such as proximity to shipping lanes and ports, as well as meteorological factors. For example, Russia received 61% of the total SOx deposition in the study area, but only 22% of the ship-originated SOx deposition fell on Russia. The share of the ship-originated SOx deposition was 0.8% in Russia, and in Poland, it was even less, 0.6%. The highest shares of ship-originated SOx were in Denmark and Estonia, approximately 7%. In 2016, the relative proportions of ship-originated SOx deposition remained rather similar for most of the countries, probably also due to the averaged meteorology that was applied in modelling.

**Table 2:** Atmospheric deposition of sulphur in the study area in 2014 and 2016, respectively, calculated using the average meteorology of the years 2014–2016. TOT is the total of dry and wet depositions. Depositions from Baltic Sea shipping are included in TOT, but the individual contributions are also shown separately as SHIP. SHIP/TOT is the share of the ship-originated SOx deposition of the total deposition. *European side of Russia. **The sea areas of the Baltic Sea

	SOx deposition in 2014					SOx deposition in 2016					CHANGE of SOx deposition from 2014 to 2016			
Country	TOT kt	TOT %	SHIP kt	SHIP %	SHIP/TOT	TOT kt	TOT %	SHIP kt	SHIP %	SHIP/TOT	TOT kt	TOT %	SHIP kt	SHIP %
Denmark	12 902	0.7	982	2.6	7.6	10 802	0.7	99	2.3	0.9	− 21	− 16.3	− 883	− 89.9
Estonia	10 841	0.6	776	2.0	7.2	9344	0.6	84	1.9	0.9	− 1497	− 13.8	− 692	− 89.2
Finland	47 501	2.7	2666	7.0	5.6	42 455	2.6	293	6.7	0.7	− 5046	− 10.6	− 2373	− 89.0
Germany	139 157	7.9	125	3.3	0.9	129 152	8.0	148	3.4	0.1	− 10 005	− 7.2	− 1102	− 88.2
Latvia	17 046	1.0	771	2.0	4.5	14 968	0.9	93	2.1	0.6	− 2078	− 12.2	− 678	− 87.9
Lithuania	22 732	1.3	572	1.5	2.5	2014	1.2	61	1.4	0.3	− 2592	− 11.4	− 511	− 89.3
Norway	55 911	3.2	692	1.8	1.2	54 473	3.4	98	2.3	0.2	− 1438	− 2.6	− 594	− 85.8
Poland	203 261	11.6	1259	3.3	0.6	183 611	11.3	300	0.7	0.2	− 1965	− 9.7	− 1229	− 97.6
Russia*	1 076 243	61.4	8445	22.2	0.8	1 022 069	63.0	1028	23.7	0.1	− 54 174	− 5.0	− 7417	− 87.8
Sweden	56 951	3.3	3786	9.9	6.6	50 163	3.1	436	10.0	0.9	− 6788	− 11.9	− 335	− 88.5
Baltic Sea**	109 299	6.2	16 869	44.3	15.4	86 106	5.3	1975	45.5	2.3	− 23 193	− 21.2	− 14 894	− 88.3
Total	1 751 844	100.0	38 068	100.0	2.2	1 623 283	100.0	4345	100.0	0.3	− 128 561	− 7.3	− 33 723	− 88.6

The Baltic Sea itself received 44–45% of the ship-originated SOx deposition both in 2014 and 2016. It was 15% of the total deposition of SOx in 2014 and 2.3% in 2016 (Table 2). The total deposition of SOx decreased by approximately 21% in the sea areas, which was more than in the riparian countries. Indeed, the spatial pattern of the ship-originated SOx deposition followed the patterns of the most heavily trafficked shipping lanes (Jonson et al. 2019). The SOx deposition was considerably larger in 2014 than in 2016, reaching far into the land areas (Jonson et al. 2019).

The current contribution of Baltic Sea shipping to the total deposition of sulphur in the BSR is very low. In 2016, the model calculations show that the total sulphur deposition from Baltic Sea shipping in the study area decreased by 7.3% due to the regulations. The share of the ship-originated SOx deposition decreased by over 88%. In all the countries in the study area, the SOx deposition from the Baltic Sea shipping decreased by over 85% and in Poland by over 97% (Table 2).

The total NOx deposition was almost the same in 2014 and 2016 (Jonson et al. 2019, Table 3), and the ship-originated NOx deposition was 2.6% of the total deposition in both years. In Lithuania and Norway, the amount of NOx depositions from the Baltic Sea shipping increased slightly. In Finland, Estonia, Sweden, and the Baltic Sea, the share of ship-originated NOx of the total deposition was the highest, approximately 7–9%. The lowest depositions were in Germany, Poland, and Russia, approximately 0.5–2%.

**Table 3:** Atmospheric deposition of nitrogen in the study area in 2014 and 2016, respectively, calculated with average meteorology of the years 2014–2016. TOT is the total of dry and wet depositions. Depositions from Baltic Sea shipping are included in TOT, but the individual contributions are also shown separately as SHIP. SHIP/TOT is the share of the ship-originated NOx deposition of the total deposition. *European side of Russia. **The sea areas of the Baltic Sea

	NOx deposition in 2014					NOx deposition in 2016					Change of NOx deposition from 2014 to 2016			
Country	TOT kt	TOT %	SHIP kt	SHIP %	SHIP/TOT	TOT kt	TOT %	SHIP kt	SHIP %	SHIP/TOT	TOT kt	TOT %	SHIP kt	SHIP %
Denmark	48 627	1.6	1822	2.3	3.7	47 391	1.6	1804	2.3	3.8	− 1236	− 2.5	− 18	− 1.0
Estonia	24 167	0.8	2193	2.8	9.1	23 605	0.8	2102	2.7	8.9	− 562	− 2.3	− 91	− 4.1
Finland	84 801	2.8	7171	9.1	8.5	82 505	2.7	6871	8.9	8.3	− 2296	− 2.7	− 3	− 4.2
Germany	588 236	19.3	2655	3.4	0.5	577 506	19.1	2586	3.3	0.4	− 1073	− 1.8	− 69	− 2.6
Latvia	41 334	1.4	2365	3.0	5.7	40 455	1.3	2311	3.0	5.7	− 879	− 2.1	− 54	− 2.3
Lithuania	54 368	1.8	1764	2.2	3.2	53 384	1.8	1770	2.3	3.3	− 984	− 1.8	6	0.3
Norway	67 491	2.2	2022	2.6	3.0	67 761	2.2	2059	2.7	3.0	27	0.4	37	1.8
Poland	376 426	12.3	4590	5.8	1.2	366 571	12.1	4560	5.9	1.2	− 9855	− 2.6	− 3	− 0.7
Russia*	1 429 360	46.8	29 156	36.9	2.0	1 428 733	47.4	28 207	36.4	2.0	− 627	0.0	− 949	− 3.3
Sweden	132 100	4.3	9387	11.9	7.1	129 422	4.3	9323	12.0	7.2	− 2678	− 2.0	− 64	− 0.7
Baltic Sea**	204 659	6.7	15 947	20.2	7.8	199 914	6.6	15 801	20.4	7.9	− 4745	− 2.3	− 146	− 0.9
Total	3051 569	100.0	79 072	100.0	2.6	3 017 247	100.0	77 394	100.0	2.6	− 34 322	− 1.1	− 1678	− 2.1

The spatial patterns of the ship-originated NOx deposition were rather similar in 2014 and 2016 (Jonson et al. 2019). The deposition of NOx was highest in the narrow zones on the landward side of the coastlines and decreased moving inland. Slightly higher depositions were observed in the central Baltic Sea as well as in the Gulf of Finland where the ship traffic is the heaviest.

### Critical load exceedances

**Fig. 2:**
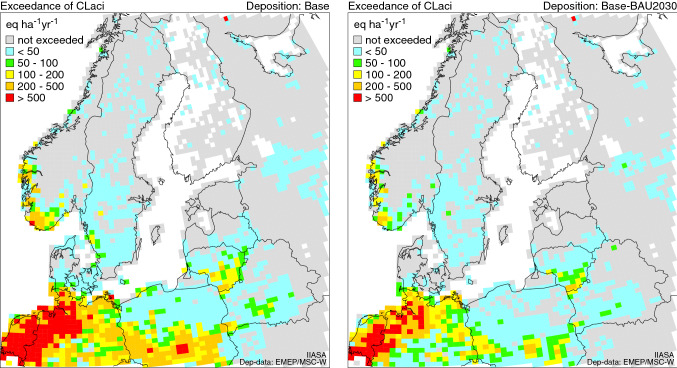
Exceedance of critical loads of acidification (CLaci) under the 2016 and 2030 base scenarios. Exceedances are displayed as average accumulated exceedance (AAE) in every 0.50° × 0.25° grid cell

Clearly, the tightening of sulphur regulations has led to a decrease in the exceedance of critical loads of acidification (Fig. 2, Table 4). After the 2015 regulation, the contribution from Baltic shipping to the exceedance of CL for acidification is very small. The land-based sources are dominating the effects. The highest exceedances were found in Germany.

**Table 4:** Exceeded area (Exarea; in percent of the total ecosystem area given in column 2) and exceedance (AAE; in eq/ha/a) for critical loads of *acidification* for the five deposition scenarios in the countries bordering the Baltic Sea (including Norway). DE:Germany, DK:Denmark, EE:Estonia, FI:Finland, LT:Lithuania, LV:Latvia, NO:Norway, PL:Poland, RU:Russia, SE:Sweden

Scenario		Base		NoBl		Bl2014		Base-BAU2030		NoBl-BAU2030	
	Ecoarea (km^2^)	Exarea (%)	AAE (eq/ha/a)	Exarea (%)	AAE (eq/ha/a)	Exarea (%)	AAE (eq/ha/a)	Exarea (%)	AAE (eq/ha/a)	Exarea (%)	AAE (eq/ha/a)
DE	106 870.5	44.1	246.5	43.9	244.1	44.2	247.4	24.4	100.2	24.3	99.1
DK	5692.3	11.9	14.3	6.7	9.1	13.7	18.0	1.4	3.0	1.2	2.6
EE	27 229.7	0.1	0.1	0.1	0.1	0.1	0.1	0	0	0	0
FI	286.0	0.7	0.4	0.6	0.3	0.7	0.4	0.6	0.3	0.6	0.3
LT	22 197.8	28.4	83.2	27.9	78.1	28.6	84.6	25.2	44.6	24.8	42.2
LV	36 630.2	3.7	3.4	2.8	2.8	3.8	3.6	1.8	1.4	1.7	1.2
NO	320 449.3	11.3	20.2	10.9	19.0	11.4	20.6	8.5	11.4	8.3	11.0
PL	96 845.7	32.9	120.2	32.3	117.6	33.1	121.0	15.0	34.3	14.8	33.6
RU	624 631.4	1.6	1.7	1.4	1.7	1.7	1.8	1.0	1.5	1.0	1.5
SE	395 225.1	5.2	3.6	5.1	3.2	5.7	4.1	3.8	1.9	3.8	1.8
All	1 636 058.0	9.4	30.0	9.1	29.2	9.6	30.3	5.9	12.5	5.8	12.2

**Fig. 3:**
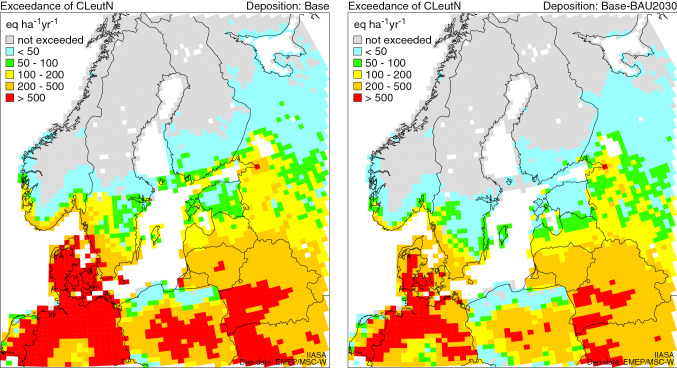
Exceedance of critical loads of eutrophication (CLeutN) under the 2016 and 2030 base scenarios. Exceedances are displayed as average accumulated exceedance (AAE) in every 0.50° × 0.25° grid cell. Note: Exceedances of eutrophication CLs are computed by using total N deposition, whereas for exceedances of acidity CLs, both total S and N deposition are needed, in both cases considering also land cover

**Fig. 4:**
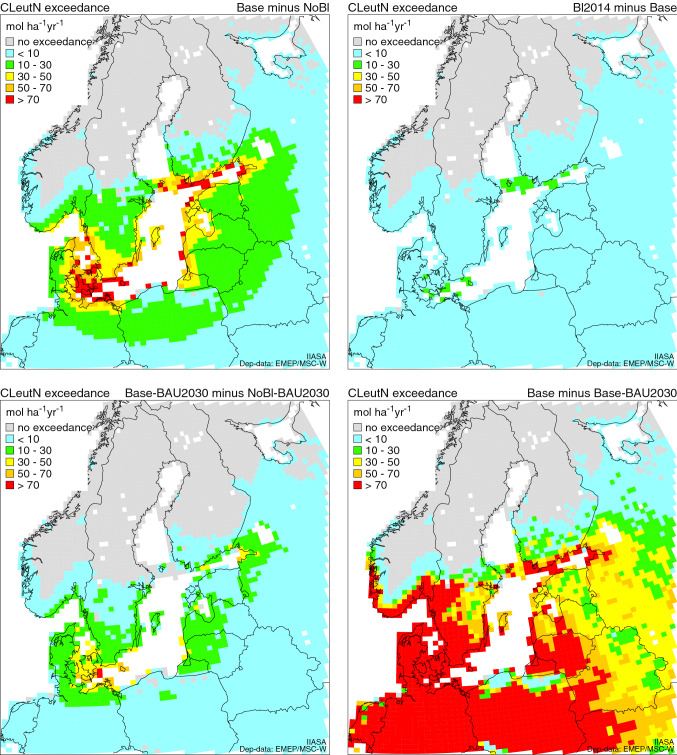
Maps of differences in CLeutN exceedances (AAE) between four combinations of the scenarios (scenario names on the upper right of each map). Note: ‘no exceedance’ means that the CLs in that grid cell are not exceeded by either deposition

For the NOx effect on eutrophication, the effect of Baltic shipping still remains considerable (Fig. 3, Table 5). The highest exceedances were in Denmark and Germany. In the 2030 scenarios, the exceedances will be slightly reduced (Fig. 4, Table 5). In the future, the NECA regulations will continue to reduce the exceedances, however, only gradually as the TIER 3 only applies to newbuildings and retrofits.

**Table 5:** Exceeded area (Exarea; in percent of the total ecosystem area given in column 2) and exceedance (AAE; in eq/ha/a) for critical loads of *eutrophication* for the five deposition scenarios in the countries bordering the Baltic Sea (including Norway). DE: Germany, DK:Denmark, EE:Estonia, FI:Finland, LT:Lithuania, LV:Latvia, NO:Norway, PL:Poland, RU:Russia, SE:Sweden

Scenario		Base		NoBl		Bl2014		Base-BAU2030		NoBl-BAU2030	
	Ecoarea (km^2^)	Exarea (%)	AAE (eq/ha/a)	Exarea (%)	AAE (eq/ha/a)	Exarea (%)	AAE (eq/ha/a)	Exarea (%)	AAE (eq/ha/a)	Exarea (%)	AAE (eq/ha/a)
DE	106 870.5	77.4	623.8	77.3	618.4	77.4	623.9	67.2	365.6	67.2	362.7
DK	5692.3	100.0	653.5	100.0	607.7	100.0	655	100.0	468.8	100.0	446.7
EE	27 229.7	74.8	58.9	59.0	33.2	75.1	60.3	41.9	23.4	29.8	18.0
FI	41 068.5	6.0	2.9	3.2	1.1	6.1	3.0	1.4	0.6	0.8	0.3
LT	22 197.8	99.2	388.5	98.9	366.9	99.2	388.4	97.8	284.1	97.6	273.1
LV	36 630.2	93.9	173.7	91.6	148.5	93.9	174.3	87.8	124.6	83.4	113.7
NO	302 948.7	11.2	18.8	10.5	16.7	11.1	18.7	6.4	7.1	6.0	6.5
PL	96 845.7	70.1	289.0	69.0	280.4	70.1	289.1	55.7	150.4	55.0	147.1
RU	624 631.4	46.2	67.7	44.6	63.2	46.2	67.9	41.3	50.7	40.1	48.5
SE	56 674.5	11.0	23.5	10.1	19.7	10.9	23.5	9.6	13.3	9.2	11.7
All	1 320 789.0	42.7	124.5	41.2	118.8	42.7	124.6	36.3	77.6	35.2	75.1

### Monetary valuation of the ship-originated SOx and NOx

The decrease in ship-originated SOx deposition from 38 kt to 3.4 kt also decreased the monetary costs of the ecosystem impacts by nearly 8 million USD, according to the STEPWISE2006 model (Tables 6, 7). The mean values of the three impact categories from the summary by Turner et al. (2004) gave higher estimates for the monetary savings due to SECA. The SO_2_ equivalent value for impacts of acidification from Ecovalue08 were even higher estimates, over 130 million USD. The prevention cost model gave the highest cost estimations, 417 million USD in 2014 and 47 million USD in 2016. Of all of these models, the Ecovalue08 is the most suitable for our purposes as it takes into account all the impacts and is more conservative than the prevention cost method. The Baltic seascape as an entity returned most of the benefits, due to its large size and proximity to shipping lanes (Table 6). In terrestrial areas, Russia, Sweden, and Finland benefitted the most (Table 1).

**Table 6:** Monetary valuation of the change in environmental impacts regarding SOx deposition from Baltic Sea shipping in 2014 and 2016 and the change from 2014 to 2016. Three sets of values for environmental impacts were applied: A, B, and C (see Table 1 for the composition of the monetary values). The results are presented in thousand USD_2018_. A is the ecosystem impacts alone from STEPWISE2006. B consists of the sum of values from the impact categories of agriculture, buildings, and ecosystems from Turner et al. (2004). C is the SO_2_ equivalent values of acidification of Ecovalue08 from Ahlroth & Finnveden (2011). D is the SOx equivalent values of prevention of acidification from Vogtländer and Bijma (2000). *European side of Russia (See Fig. 1). **The sea areas of the Baltic Sea

Country	A 2014, k$	A 2016, k$	A change 2014 to 2016, k$	B 2014, k$	B 2016, k$	B change 2014 to 2016, k$	C 2014, k$	C 2016, k$	C change 2014 to 2016, k$	D 2014, k$	D 2016, k$	D change 2014 to 2016, k$
Denmark	231	23	− 208	1342	135	− 1206	3795	383	− 3413	10 771	1086	− 9685
Estonia	183	20	− 163	1060	115	− 945	2999	325	− 2675	8511	921	− 7590
Finland	628	69	− 559	3642	400	− 3242	10 304	1132	− 9171	29 241	3214	− 26 027
Germany	294	35	− 259	1708	202	− 1506	4831	572	− 4259	13 710	1623	− 12 087
Latvia	182	22	− 160	1053	127	− 926	2980	359	− 2620	8456	1020	− 7436
Lithuania	135	14	− 120	781	83	− 698	2211	236	− 1975	6274	669	− 5605
Norway	163	23	− 140	945	134	− 812	2675	379	− 2296	7590	1075	− 6515
Poland	297	7	− 289	1720	41	− 1679	4866	116	− 4750	13 809	329	− 13 480
Russia*	1989	242	− 1747	11 538	1404	− 10 133	32 639	3973	− 28 666	92 625	11 275	− 81 350
Sweden	892	103	− 789	5172	596	− 4577	14 633	1685	− 12 947	41 525	4782	− 36 743
Baltic Sea**	3973	465	− 3508	23 046	2698	− 20348	65 197	7633	− 57 564	185 019	21 662	− 163 358
Total	8967	1023	− 7943	52 008	5936	− 46072	147 129	16 793	− 130 336	417 530	47 656	− 369 874

**Table 7:** Monetary valuation of the change in environmental impacts regarding NOx deposition from Baltic Sea shipping in 2014 and 2016 and the change from 2014 to 2016. See Table 1 for the composition of the monetary values. E consists of the sum of values from the impact categories of agriculture, buildings, ecosystems and fertilisation effects from Turner et al. (2004) and Weidema (2009). Results are presented in USD_2018._ *European side of Russia. **The sea areas of the Baltic Sea

Country	D 2014, k$	D 2016, k$	D change from 2014 to 2016, k$
Denmark	3147	3116	− 31
Estonia	3788	3631	− 157
Finland	12 387	11 869	− 518
Germany	4586	4467	− 119
Latvia	4085	3992	− 93
Lithuania	3047	3057	+10
Norway	3493	3557	+64
Poland	7929	7877	− 52
Russia*	50 363	48 724	− 1639
Sweden	16 215	16 104	− 111
Baltic Sea**	27 546	27 294	− 252
Total	136 586	133 688	− 2899

The monetary values of the impact categories of NOx decreased by 2% (136 to 133 million dollars) between the years 2014 and 2016 (Table 7). Russia benefited the most from this small improvement.

## Discussion

### The reduction in sulphur deposition

Sulphur emissions originate from several sources mainly from industry and traffic, of which shipping is one part. Industry and land-based traffic have successfully decreased their emission. This has greatly reduced the size of the area in Europe where sulphur deposition exceeds critical loads, but shipping has been lagging behind (Amann et al. 2011). The implementation of the IMO MARRPOL Annex VI regulations has led to a steep decrease in SOx emissions from shipping (Johansson et al. 2013; Jonson et al. 2015; IMO 2017; HELCOM 2018 pp. 41, 42).

The direct environmental impacts of SOx are related to acidification. Acidification has different impacts on the natural environment in soils (van Breemen et al. 1982), freshwaters (Krzyzanowski and Innes 2010), oceans, and brackish waters. In particular, calcifying species in the sea are under threat (Azevedo et al. 2015). However, ocean acidification is mostly linked to rising CO_2_ levels, and oceans are generally well buffered against sulphur acidification (LeDuc et al. 2014; Stips et al. 2016). The SOx emissions from shipping, however, form strong acids and, thus, may cause pH reductions of the same order of magnitude as the weak carbonic acid formed from CO_2_ acidification in ocean waters, especially in heavily trafficked areas (Hassellöv et al. 2013; Stips et al. 2016). Still, in the Baltic Sea, the long-term effects of ship-borne acid deposition, including scrubber wash water, on pH and alkalinity should be small, especially in the surface waters (Turner et al. 2018). This is because the Baltic Sea surface waters are exported to the North Sea (Turner et al. 2018). The Baltic Sea is predicted to be warmer, less saline, and more acid in the future due to climate change and cumulative impacts of other stressors (Jutterström et al. 2014), of which shipping is one. A decrease in and lower levels of ship-originated SOx in order to avoid negative ecosystem developments have been achieved by regulations, but the scrubber wash waters also need to be regulated. The non-linear dynamics in marine ecosystems call for precautions in management (Scharin et al. 2016). In addition to the impacts on ecosystems, SOx damages buildings and cultural heritage that are part of the monetisation models.

If the critical loads of acidification are exceeded, it may decrease biodiversity, which holds value for humans in many ways. From a management point of view, a safe strategy seems to be to require a minimum level of biodiversity for any ecosystem to be sustained (Arrow et al. 1996). The central question in valuing biodiversity has been whether we should value all elements of biodiversity (e.g. the existence of species, the resilience of communities, etc.) in monetary terms or whether they should possess an intrinsic value regardless of human benefit (Nunes and van den Bergh 2001).

### Monetisation of the deposition of ship-originated SOx

The monetisation analysis of SOx and NOx in the Baltic Sea Region covers terrestrial and sea areas including land, lake and brackish water areas. Considering the large geographical area, and differences in the effects on different waterbodies and land sites, it is clear that using a single coefficient to cover it all is not very accurate. On the other hand, conducting a finer scale analysis on this large area is practically impossible. In this study, we have carried out the best possible large-scale analysis with current monetisation values. The environmental effects of strengthening the SECA have not been analysed before. The analysis was necessary in order to compare the costs of regulation to the benefits by using the same kind of methodology as was being used in an ex-ante analysis. For this discussion, we bring in the environmental benefit of 130 million USD. This can be compared to the costs of compliance of 662 million dollars (Repka et al. 2019), and the health benefits based on decreased mortality of at least 557 million dollars (Barregård et al. 2019). Thus, the health and environmental benefits of the regulations exceed the control costs.

### Nitrogen deposition

A significant amount of nitrogen still originates from the shipping in the Baltic Sea. High levels of NOx depositions also extend into northern Germany and Denmark. The maximum deposition from the Baltic Sea shipping is along the coastlines rather than in the shipping lanes where ammonium nitrate is formed in combination with ammonia that mainly comes from agriculture (Jonson et al. 2019). In many coastal areas of the Baltic Sea, more than 10% of the total amount of nitrogen deposition is due to shipping. Typically, dry depositions on subgrid-scale forest ecosystems (both coniferous and deciduous) are higher than the grid average.

The deposition of nitrogen has been estimated to have a larger impact on the terrestrial environment than SOx, which after 2015 was mostly concentrated in the sea areas. Compared with the STEPWISE2006 model, values for eutrophication from the other models for the valuation of N are almost three times higher; however, it should be noted that they all have different methods and scopes (Pizzol et al. 2015).

Excess nitrogen causes eutrophication in freshwater and affects species decomposition on terrestrial ecosystems leading to loss of biodiversity (e.g. Rabalais 2002; Stevens et al. 2010); this is supported by the results of the critical load analysis which shows that the CLs are exceeded in the southern part of the BSR and this will continue in the future. Thus, more stringent regulation is needed, and in 2021, the NECA will be tightened (IMO 2017). Since the volume of maritime traffic has remained approximately the same and NOx emissions have even increased in some areas, this shows that the current level of NOx regulation is not sufficient, mainly because it only concerns new ships (Fig. 4). However, it will be more successful in coming years when the fleet is renewed with TIER III vessels.

## Conclusions

In policy assessments, all categories of environmental and health aspects should be included and not just human health (Lähteenmäki-Uutela et al. 2017). This is the first attempt to monetarise the environmental benefits of SECA regulation, and we noticed that there is a lot of uncertainty in the monetarization methods. There are no good estimates for the acidification of the Baltic Sea, and we were forced to use the same coefficients as for land areas. Another message is the need to develop valuation of different types of ecosystems, as it is needed in political discussion of environmental protection.
